# Effects of an Integrated Health Care Program for Children

**DOI:** 10.20463/jenb.2016.0037

**Published:** 2017-03-31

**Authors:** Ok Hyun Kim, Jin Kyung Park

**Affiliations:** 1 Department of Food & Nutrition, College of Natural Sciences, Seoul Women’s University, Seoul Republic of Korea; 2 Department of Sports & Health Care, SangMyung University, Seoul Republic of Korea

**Keywords:** Integrated health care program, Physical fitness, Dietary habits, Psychological changes

## Abstract

**[Purpose]:**

This study examined the effects of an integrated health care program in elementary school students.

**[Methods]:**

The integrated program comprised exercises (3–4 times/week) and six sessions on nutritional and psychological education. Anthropometric measurements were recorded before the intervention. Additionally, physical fitness, dietary habits, nutrition knowledge, and psychological changes were assessed before and after the program.

**[Results]:**

In total, 29% of the subjects were overweight and obese before the intervention (32% boys and 26% girls). There was a significant increase in flexibility, endurance, and cardiovascular endurance after the implementation of the program. Additionally, as a result of the program, participants showed improvement in nutrition knowledge and dietary habits. After the training, children tended to exhibit increased self–efficacy and lower stress, but the findings were not statistically significant.

**[Conclusion]:**

Implementation of an integrated health care program for the prevention and treatment of obesity could have a positive impact on children’s health. It is hoped that continued research on the long-term effects of such programs is conducted along with the development of various programs.

## INTRODUCTION

Childhood, which encompasses elementary school age, is a critical period for physical and mental growth and development as well as for character building. Further, this is a period in which dietary habits and physical activity habits – the factors that affect current and future health status – are established, based on which children build physical strength that serves as the basis for lifelong health^1^. 

According to the Ministry of Education, over the past 15 years (1999–2013), the average height and weight of 10-year-old Korean boys increased by 1.1 cm (from 141.6 cm to 142.7 cm) and 3.7 kg (from 36.5 kg to 40.2 kg), respectively, and those of 10-year-old Korean girls increased by 0.8 cm (from 142.4 cm to 143.2 cm) and 2.3 kg (from 36.1 kg to 38.4 kg), respectively. However, in the same period, physical fitness declined in both groups^2^. In addition, multiple studies have raised concerns regarding children, including reduced physical activity, nutritional imbalance, increased obesity rate, school maladaptation, depression, and school violence, all of which threaten children’s health. 

The World Health Organization (WHO) defines health as a state of complete physical, mental, and social well-being and not merely the absence of disease and infirmity^3^. Thus, a balance among adequate physical activities, nutritional balance, and psychological stability by reducing stress must be achieved to maintain health. 

Most health-related programs for children in Korea aim at providing secondary treatment for a subset of health-risk groups, such as reducing obesity for obese children, instead of offering a comprehensive health management for the prevention of obesity and diseases^4, 5^. Further, only a handful of the programs comprise independent sessions of exercise as well as nutritional and psychological education or combined sessions of exercise and nutritional education^5-7^. Therefore, the present study sought to identify the effects of a 10-week, integrated physical activity program, consisting of both exercise and psychological education, on the physical fitness, nutritional knowledge, and dietary habits, as well as on the psychological changes (self-efficacy and stress) in the fifth grade participants. 

## METHODS

### Study Participants

Fifty-two fifth grade students (two classes) from anelementary school in Seongbuk-gu, Seoul, were enrolledin the study after obtaining consent from the school andtheir parents. With cooperation from the school, the programwas administered in the playground, auditorium,and lecture halls during 3–4 hours of physical educationsessions allotted for the study. 

### Content and Methods

#### Program composition

The integrated program comprised 36 sessions of physical activity and six sessions of nutritional and psychological education, each implemented for 10 weeks, from October to December, 2013. 

#### Physical activity program

The physical activity portion of the program comprised circulation exercise and new sports exercise, mostly based on aerobic exercise and muscle strengthening exercise, to boost the physical strength of schoolaged children. The initial, development, and maintenance stages involved low-intensity exercise (40–50% maximal heart rate [HRmax]), moderate-intensity exercise (50–60% HRmax, and moderate and high-intensity exercise (60–80% HRmax), respectively. To keep the children interested, the exercise program consisted of bowling, basketball, badminton-like program, floorball, and T-ball baseball, in addition to muscle-strengthening exercise, ranging from light muscle strengthening to heavier training using equipment. 

#### Nutritional education

With a focus on achieving a balanced diet through appropriate diet and weight management with lower sodium intake, the nutritional education component of the program comprised lectures on concepts of health (importance of health, and health and nutrition), evaluation of nutritional status (survey of dietary habits and nutrition intake), functions and roles of nutrients (explanation of the food cycle), appropriate dietary habits (importance of breakfast and healthy snacks), reading nutrition facts, and reducing salt intake (sodium content of each food and saltiness test). 

#### Psychological education

The psychological education component of the program comprised lectures on the importance of mental health, evaluation of psychological state psychological survey), boosting self-respect methods of communication, improving leadership, stress relief (meditation and five-senses therapy), and depression prevention (). 

### Assessment of the effects of the intervention

#### Anthropometric measurements

Prior to the program, the participants’ anthropometric measurements were taken; height was measured using a height scale, and body weight and body fat percentage were measured using InBody 7.0 (Biospace, Korea). The children were classified into underweight (<5th percentile), normal weight (5th percentile–85th percentile), overweight (85th percentile–95th percentile), and obese (>95th percentile or body mass index [BMI] >25) based on the standard percentages of BMI for age and sex as per the growth chart for children and adolescents developed by the Centers for Disease Control and the Korean Pediatric Society. 

#### Physical fitness measurement

To assess the effects of the intervention in improving students’ physical fitness, flexibility (sit and reach), cardiovascular endurance (round-trip long run), muscular endurance (sit and rollup), and power (50-m run) were measured twice, once before and once after the intervention. 

#### Nutritional knowledge and dietary habit

To assess the effects of the intervention on nutritional education, changes in students’ nutritional knowledge and dietary habits were surveyed before and after the intervention. Educational materials that we developed as well as data provided from some nutrition-related websites were used for the nutritional education component, and educational sessions included various activities, including lectures, group discussions, and games to capture students’ attention and promote active participation. 

① Nutritional knowledge: Nutritional knowledge was measured with 10 items on health, nutrients, and sodium intake. Students were given a score of 10 for a correct answer and a score of 0 for a wrong answer. The mean score for the 10 items was used. All the questions were based on the contents of the nutritional education. 

② Survey of dietary habits: A 20-item scale developed by the Korean Society for Health Promotion and Disease Prevention was used to survey dietary habits. For items on desirable dietary habits, a score of 1, 3, and 5 were given for “0–2 days,” “3–5 days,” and “6–7 days,” respectively; the scores were reversed for items on undesirable dietary habits. The items pertained to skipping breakfast; extent of consumption of fruits, vegetables, fast food, ramen, soft drinks, and milk intake; and appropriate dietary habits^9, 10^. 

#### Assessment of psychological improvement

The psychological changes produced by the intervention were assessed using psychological tests before and after the intervention. Items pertaining to self-efficacy, leadership life skills, and daily stress were included in the test. 

① Self-efficacy: Self-efficacy was measured using the self-efficacy scale originally developed by Sherer et al. (1982) and translated and validated by Kim (1997) for use with Korean adolescents^11, 12^. The scale comprises 17 items pertaining to the perception of individual skills that affect outcomes in a variety of situations, and each item is measured on a five-point scale. 

② Leadership life skills: The leadership life skills questionnaire for elementary school children developed by Jin (2006) was used to measure leadership life skills^13^. This 30-item scale comprises five categories of skills related to interacting with others, learning skills, skills in understanding oneself, skills in working with groups, and decision-making skills, and each item is measured on a five-point scale. 

③ Daily stress: The daily stress scale for Korean children developed by Han and Yoo (1995) was used to measure daily stress14. This 42-item scale comprises six categories of stress related to parents, family background, friends, studies, teachers and school, and environment, and each item is measured on a five-point scale. 

### Data processing

All values were presented as mean ± standard deviation (SD). All statistical analyses were performed using the SAS software program (version 9.2; SAS Institute Inc., Cary, NC, USA), with a significance level of α < 0.05. Sex-specific differences were analyzed using the Chi-square test and student t-tests, and post-intervention changes were measured using paired t-tests. 

## RESULTS

### Study Participants

Fifty-two students were enrolled in this study, of whom 25 were boys (48.1%) and 27 were girls (51.9%). The mean height and weight of the boys and girls was 148.5 cm and 42.4 kg and 146.9 cm and 39.1 kg, respectively, showing no significant differences between the sexes. There were no significant sex differences in BMI, but body fat percentage was higher in girls. Further, there were no significant sex differences in the proportion of obesity, with 17 normal weight (68%), six overweight (24%), and two obese (8%) boys, and 20 normal weight (74.1%), five overweight (18.5%), and two obese (7.4%) girls (Table 1). 

**Table 1:** Distribution of the subjects

Variables		Boys	Girls	P-value
N(%)^*^		25 (48.1%)	27 (51.9%)	
Height (cm)^†^		148.5 ± 7.1	146.9± 7.5	0.4326
Weight (kg)		42.4 ± 10.6	39.1 ± 7.1	0.1884
Body mass index		19.0 ± 3.6	18.0 ± 2.4	0.2422
Percent fat		11.7 ± 7.4	18.7 ± 5.8	0.0004
	Underweight	0 (0.0)	0 (0.0)	0.8971
	Normal	17 (68.0)	20 (74.1)	
Obesity	Overweight	6 (24.0)	5 (18.5)	
degree^‡^	Obese	2 (8.0)	2 (7.4)	

^*^ Values are n (%)

^†^ Values are mean±SD.

*P*-value by Chi-square test or student *t*-test.

^‡^ Percentile of the sex-specific BMI for age growth charts: underweight (BMI <5^th^ percentile), normal weight (5^th^ percentile ≤ BMI < 85^th^ percentile), overweight (85^th^ percentile ≤ BMI < 95^th^ percentile), obesity ( 95^th^ percentile ≤ BMI or 25 ≤ BMI)

### Pre- and Post-intervention Assessment Changes in physical fitness

Changes in physical fitness after the intervention are illustrated in Table 2. For boys, all the parameters of physical fitness, with the exception of power (i.e., flexibility, muscular endurance, and cardiovascular endurance), improved significantly after completing the program. For girls, all the parameters of physical fitness (i.e., flexibility, muscular endurance, power, and cardiovascular endurance) improved significantly after completing the program. Particularly, flexibility scores were higher in girls than in boys. Further, girls’ pre-program muscular endurance was lower than that of boys, but it increased to similar levels after the program. 

**Table 2:** Change of physical fitness at pre and post-intervention

Variables	Boys			Girls		
	Pre(n=24)	Post(n=24)	P-value	Pre(n=25)	Post(n=25)	P-value
Sit & reach (cm)^*^^†^	8.0 ± 4.7	8.8 ± 4.6	0.0124	12.7 ± 5.6	13.4 ± 5.3	0.0019
Sit & Roll ups (times)^‡^	63.5 ± 33.5	73.1 ± 32.8	0.0045	53.1 ± 26.1	72.0 ± 22.8	<.0001
50m running (sec)^§^	10.9 ± 2.5	10.4 ± 1.5	0.1088	10.2 ± 0.7	10.0 ± 0.8	0.0467
Round-trip long run (times)^¶^	66.4 ± 14.8	73.6 ± 17.0	0.0018	54.7 ± 8.3	62.9 ± 13.3	0.0002

^*^ Values are mean±SD.

*P*-value by paired *t*-test.

^†^ Sit and reach is an index flexibility.

^‡^ Sit roll up is an index muscular endurance.

^§^ 50m running is an index of power.

^¶^ Round-trip long run is an index cardiorespiratory endurance.

### Changes in dietary habits

Post-program changes in dietary habits are presented in Table 3. Boys’ dietary habit score increased from 71.4 points before the program to 75.1 points after the program, and that of girls increased from 75.2 points to 77.3 points after the program. However, these differences did not reach statistically significant levels. Most dietary habits had changed positively after participating in the program for both boys and girls. Particularly, boys showed significant improvements in “having three meals on time” and avoiding “having caffeinated drinks more than three times a day,” while girls showed significant improvements in avoiding “eating food containing cholesterol.”

**Table 3:** Change of dietary habits at pre and post-intervention

Variables	Boys			Girls		
	Pre(n=24)	Post(n=24)	P-value	Pre(n=25)	Post(n=25)	P-value
1. Have three meals on time^*^	3.8 ± 1.3	4.2 ± 1.4	0.0049	4.6 ± 0.8	4.0 *±* 1.2	0.3765
2. Have adequate amount of meals	3.9 *±* 1.3	3.9 *±* 1.3	0.3273	4.1 *±* 1.4	4.1 *±* 1.3	0.4165
3. Have protein foods(meat, fish, egg, bean, bean curd) more than two meals a day	3.8 *±* 1.4	4.2 *±* 1.2	0.8239	3.7 *±* 1.7	4.0 *±* 1.4	0.4907
4. Have green vegetables(carrot, spinach)	3.8 *±* 1.5	3.8 *±* 1.3	0.7463	3.7 *±* 1.4	4.1 *±* 1.2	0.3563
5. Have foods using plant oils	3.2 *±* 1.4	3.7 *±* 1.4	0.1701	3.6 *±* 1.6	3.6 *±* 1.5	0.8239
6. Have fruits or fruit juice(no sugar)	3.9 *±* 1.4	3.8 *±* 1.6	0.5743	4.0 *±* 1.3	4.0 *±* 1.5	0.5426
7. Drink milk or dairy foods	4.1 *±* 1.3	3.8 *±* 1.5	0.4498	3.9 *±* 1.6	3.9 *±* 1.5	0.8022
8. Have seaweeds(e.g. brown seaweed, laver, tangle)	3.3 *±* 1.5	3.6 *±* 1.5	0.1479	3.9 *±* 1.5	3.4 *±* 1.5	0.5743
9. Have meals slowly and nicely	4.1 *±* 1.2	4.1 *±* 1.2	0.7136	4.2 *±* 1.2	4.0 *±* 1.6	0.6639
10. Have meals with diverse side dishes	3.7 *±* 1.6	3.2 *±* 1.7	0.7463	3.6 *±* 1.5	3.5 *±* 1.6	0.5238
11. Have breakfast	4.0 *±* 1.6	3.6 *±* 1.7	0.8022	3.9 *±* 1.6	4.0 *±* 1.4	0.0961
12. Have processed food(ramen, snacks)	2.9 *±* 1.2	3.5 *±* 1.3	0.3273	3.2 *±* 1.0	3.6 *±* 1.1	0.4907
13. Eat out at least once a day	3.7 *±* 1.1	4.0 *±* 1.3	0.6032	3.9 *±* 1.0	4.1 *±* 1.2	0.6469
14. Have animal food or food that contains a lot of cholesterol	2.6 *±* 1.3	2.8 *±* 1.1	0.0575	3.2 *±* 1.3	3.4 *±* 1.2	0.0054
15. Have salty foods	3.6 *±* 1.4	3.8 *±* 1.5	0.6469	3.8 *±* 1.3	3.8 *±* 1.4	1.0000
16. Have sweets(sugar, chocolate, candy)	3.3 *±* 1.2	2.7 *±* 1.6	0.6032	3.2 *±* 1.4	3.2 *±* 1.4	0.0559
17. Have hot and spicy foods	3.2 *±* 1.5	3.2 *±* 1.6	0.4498	3.0 *±* 1.6	3.0 *±* 1.5	0.5381
18. Have instant foods(hamburger, pizza)	3.0 *±* 1.2	4.0 *±* 1.3	0.1344	3.4 *±* 1.5	4.4 *±* 1.2	0.0559
19. Drink beverage(coke, coffee) that has caffeine more than three a day	3.7 *±* 1.3	4.8 *±* 0.9	0.0317	4.3 *±* 1.1	4.6 *±* 1.0	0.3273
20. Have snacks before go to bed	3.6 *±* 1.4	4.5 *±* 1.0	0.3067	4.0 *±* 1.3	4.4 *±* 1.1	0.7136
Total score	71.4 *±* 11.1	75.1 *±* 11.9	0.0975	75.2 *±* 16.2	77.3 *±* 15.0	0.2924

^*^ Values are mean ± SD.

*P*-value by paired *t*-test.

### Changes in nutritional knowledge

Post-program changes in nutritional knowledge are shown in Table 4. Both the boys and girls had significant increases in nutritional knowledge after participating in the program. 

**Table 4:** Nutrition Knowledge of the subjects

Variables	Boys			Girls		
	Pre(n=24)	Post(n=24)	P-value	Pre(n=25)	Post(n=25)	P-value
Nutrition Knowledge Score^*^	75.2 ± 11.6	77.6 ± 11.3	<.0001	69.6 ± 11.4	73.6 ± 14.4	<.0001

^*^ Values are mean ± SD.

*P*-value by paired *t*-test.

### Changes in self-efficacy

Post-program changes in self-efficacy have been shown in Table 5. Both the boys and girls showed mild increases in self-efficacy after the program, but not to statistically significant levels. 

**Table 5:** Self-Efficacy of the subjects

Variables	Boys			Girls		
	Pre(n=24)	Post(n=24)	P-value	Pre(n=25)	Post(n=25)	P-value
Self-Efficacy^*^	2.7 ± 0.4	2.9 ± 0.4	0.3568	2.6 ± 0.4	2.8 ± 0.4	0.2129

^*^ Values are mean ± SD.

*P*-value by paired *t*-test.

### Changes in leadership life skills

Changes in leadership life skills have been shown in Table 6. Boys showed increased scores in all five items (interacting with others, learning skills, skills in understanding oneself, skills in working with groups, and decision- making skills) after participating in the program, and the differences in the learning skills and skills in working with groups were statistically significant. Girls either showed no changes or exhibited increased scores in all five items, but none of the differences were statistically significant. 

**Table 6:** Leadership life skills of the subjectsa

Variables	Boys			Girls		
	Pre(n=24)	Post(n=24)	P-value	Pre(n=25)	Post(n=25)	P-value
Relationship skill ^*^	4.1 ± 0.8	4.2 ± 0.7	0.3545	4.4 ±0.5	4.4 ± 0.5	0.2328
Learning ability skill	3.8 ± 1.1	4.2 ± 0.7	0.0188	3.9 ±0.7	4.1 ± 0.8	0.3095
Self-interest skill	4.2 ± 0.8	4.4 ± 0.7	0.079	4.3 ± 0.6	4.4 ± 0.5	0.2369
Group activity skill	3.9 ± 0.8	4.2 ± 0.7	0.0266	4.1 ±0.7	4.1 ± 0.8	0.697
Decision skill	3.9 ± 0.9	4.2 ± 0.9	0.1866	4.0 ±0.7	4.1 ± 0.6	0.0941

^*^ Values are mean ± SD.

*P*-value by paired *t*-test.

### Daily stress

Post-program changes in daily stress are shown in Table 7. Boys showed reduced stress in all six items, with statistically significant reductions in stress related to parents, studies, teachers and the school, and the environment. Girls either showed no changes or exhibited reductions in stress scores, but not to a statistically significant level. 

**Table 7:** Daily hassles scale of the subjects

Variables	Boys			Girls		
	Pre(n=24)	Post(n=24)	P-value	Pre(n=25)	Post(n=25)	P-value
Stress related to parents^*^	2.4 ± 0.9	2.0 *±* 1.2	0.0233	2.1 *±* 0.9	1.9 *±* 1.0	0.1283
Stress-related family background	1.7 *±* 0.7	1.5 *±* 0.8	0.2624	1.5 *±* 0.6	1.5 *±* 0.7	1.0000
Stress related to friendship	1.7 *±* 1.0	1.4 *±* 0.9	0.0574	1.5 *±* 0.8	1.5 *±* 0.6	0.5234
Stress-related studies	2.3 *±* 0.9	1.7 *±* 1.1	0.0027	2.2 *±* 1.2	1.9 *±* 1.1	0.0848
Stress-related teachers and school	1.9 *±* 1.0	1.6 *±* 0.8	0.0282	1.4 *±* 0.5	1.3 *±* 0.4	0.3290
Stress related to environment	1.9 *±* 1.1	1.6 *±* 0.9	0.0492	1.4 *±* 0.5	1.2 *±* 0.5	0.0776

^*^ Values are mean ± SD.

*P*-value by paired *t*-test.

## DISCUSSION

The objective of this study was to examine the effects of an integrated physical activity program comprising nutritional and psychological education on the physical fitness, psychological states, dietary habits, and nutritional knowledge of elementary school children. The mean height and weight for boys was 148.5 cm and 42.4 kg, respectively, and for girls was 146.9 cm and 39.1 kg, respectively; these were above the national average for Korean 10-year-olds reported by the Ministry of Education in 2013 (boys: 142.7 cm and 40.2 kg; girls: 143.2 cm and 38.4 kg)^2^. Further, there were no sex differences in BMI, but body fat percentage was significantly higher in girls than that in boys. Proportions of overweight and obese children were higher, though not statistically significantly, in boys. The proportions of obese children in this study (boys: 32.0%, girls: 25.4%) were higher than those reported by Kim et al.^15^ based on the same obesity criterion (boys: 19.1%; girls: 13.9%). 

Prior to the intervention, boys had lower flexibility and muscular endurance than the national average for Korean 10-year-olds, as reported by the 2013 Student Health and Fitness Evaluation by the Ministry of Education, but both values increased to higher levels than the national average after completing the program. Further, the participants had higher power than the national average for 10-yearolds before and after the program. Conversely, despite the fact that boys showed significant improvements in cardiovascular endurance after the program, the value was still lower than the national average cardiovascular endurance of Korean 10-year-olds. For girls, flexibility was lower than the national average before the program, but it increased to higher levels than the national average after the program. Muscular endurance was higher than the national average and it significantly increased after participation in the program. Power was similar to the national average, but decreased after participation in the program. Similar to the trend observed in boys, cardiovascular endurance significantly increased after the program, but it was still lower than the national average. In essence, the integrated program improved flexibility, muscular endurance, and cardiovascular endurance in both boys and girls, while power either remained unchanged or reduced. According to Park et al.^16^, reduced physical strength not only signifies physical problems such as increased obesity rate, but also leads to an array of social problems such as bullying, violence, suicide, game addiction, and depression. Further, they stated that regular exercise promotes physical fitness and plays a positive role in relieving physical and mental stress. 

Dietary habits in childhood may have a lasting impact on a child’s lifelong dietary habits; hence, it is critical to shape appropriate dietary habits in childhood. In our study, participation in the program led to significant improvements in nutritional knowledge, and though not statistically significant, the total dietary habit score also increased after the program. In a study that only performed four sessions of nutritional education over 4 weeks in fifth graders, children showed significant improvements in nutritional knowledge, dietary attitude, and dietary behaviors^17^. 

Many studies have suggested that nutritional education increases children’s nutritional knowledge and brings about desirable changes in dietary behaviors and dietary intake^17, 18^. However, long-term systematic nutrition education is required for increased nutritional knowledge to produce changes in dietary attitudes, behaviors, and intake, but such changes are difficult to be delivered through most of the current nutrition education programs for children, as they are generally administered via shortterm programs. 

Self-efficacy refers to the belief in one’s own capacity to perform behaviors that would bring about certain outcomes. Daily stress refers to trivial problems that most people commonly encounter in their daily lives. In Kim’s study on adolescents, self-efficacy was enhanced with advancing stage of exercise, and regular participation in exercise increased the significance of self-efficacy as a psychological variable^12^. Further, academic stress is known to have a vast impact on self-respect during childhood, the most important period in life^20^. After participating in our program, the participants’ self-efficacy improved mildly, with an overall reduction in daily stress, but not to significant levels. Therefore, we speculate that our program contributed to the positive alteration of the participants’ psychological states. 

Implementation of an integrated health management program for the prevention and treatment of obesity in childhood, a critical period that shapes a child’s lifelong health, may have a positive impact on children’s health. Hence, we hope that future studies continue to develop various programs and examine the long-term effects of programs for children. 
